# Editorial of Special Issue “Piezoelectric Transducers: Materials, Devices and Applications”

**DOI:** 10.3390/mi11070678

**Published:** 2020-07-13

**Authors:** Jose Luis Sanchez-Rojas

**Affiliations:** Microsystems, Actuators and Sensors Lab, Universidad de Castilla-La Mancha, 13071 Ciudad Real, Spain; JoseLuis.SAldavero@uclm.es

Advances in miniaturization of sensors, actuators, and smart systems are receiving substantial industrial attention, and a wide variety of transducers are commercially available or possess high potential to impact emerging markets. Substituting existing products based on bulk materials, in fields such as automotive, environment, food, robotics, medicine, biotechnology, communications, Internet of things, and related technologies, with reduced size, lower cost, and higher performance, is now possible with potential for manufacturing using advanced silicon-integrated circuits technology or alternative additive techniques from the milli- to the micro-scale.

This Special Issue has compiled a total of 34 papers focused on piezoelectric transducers, covering a wide range of topics including the design, fabrication, characterization, packaging, and system integration or final applications of milli/micro/nano-electro-mechanical systems based transducers featuring piezoelectric materials and devices.

I would like to take this opportunity to thank all the authors for submitting their papers to this Special Issue. I also want to thank all the reviewers for dedicating their effort and time in assisting to improve the quality of the submitted papers.

In view of the success reached in the number and quality of papers published, we plan to open a second volume where we hope to continue with the latest advances in piezoelectric transducers and their trend to miniaturization, efficiency, and new applications.

